# Facile and Convenient Synthesis of New Thieno[2,3-b]-Thiophene Derivatives

**DOI:** 10.3390/molecules15129418

**Published:** 2010-12-20

**Authors:** Yahia Nasser Mabkhot, Nabila Abd Elshafy Kheder, Abdullah Mohammad Al-Majid

**Affiliations:** 1 Department of Chemistry, Faculty of Science, King Saud University, P. O. Box 2455, Riyadh 11451, Saudi Arabia; 2 Department of Chemistry, Faculty of Science, Cairo University, Giza 12613, Egypt

**Keywords:** thieno[2,3-b]thiophene, bis-heterocycles, nucleophilic addition, 2-bromo-1-phenylethanone

## Abstract

A facile and convenient synthesis of bis(2-(1H-benzo[d]imidazol-2(3H)-ylidene)-3-oxopropanenitrile), bis((3-amino-5-(methylthio)-1H-pyrazol-4-yl)methanone) and bis(2-thioxo-1,2-dihydropyrimidine-5-carbonitrile) derivatives incorporating a thieno- [2,3-b]thiophene moiety *via* versatile, readily accessible diethyl 3,4-dimethylthieno-[2,3-b]thiophene-2,5-dicarboxylate (**1**) is described.

## 1. Introduction

Recently, bis(heterocycles) have received a great deal of attention, not only as model compounds for main chain polymers but also because many biologically active natural and synthetic products have molecular symmetry [1,2,3,4,5,6,7,8]. On the other hand, many thiophene-containing compounds, including annulated compounds, exhibit biological activities [9,10]. Thienothiophene derivatives have been developed for different purposes in the pharmaceutical field and have been tested as potential antitumor, antiviral and antibiotic, antiglaucoma drugs, or as inhibitors of platelet aggregation [11,12,13,14,15]. In addition, thienothiophenes have potential applications in a wide variety of optical and electronic systems [16,17,18]. Encouraged by all these findings and in continuation of our ongoing research program investigating the utilisation of compound **1** as a versatile and useful building block for the synthesis of a wide variety of bis-heterocyclic systems [19,20], we report herein a convenient route to some novel bis-heterocycles derivatives.

## 2. Results and Discussion

**Scheme 1:**
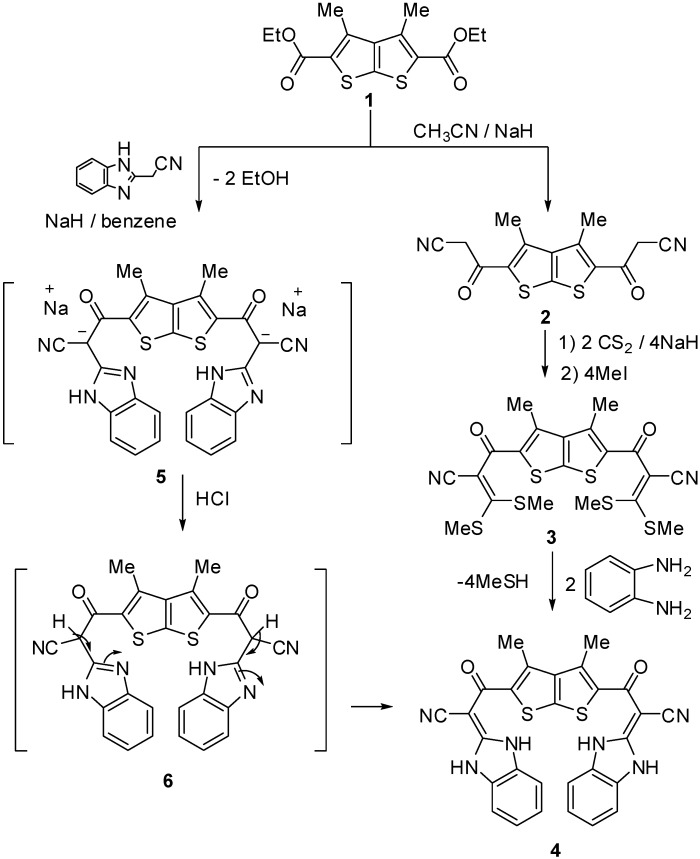
Synthesis of 3,3'-(3,4-dimethylthieno[2,3-b]thiophene-2,5-diyl)bis(2-(1H-benzo[d]imidazol-2(3H)-ylidene)-3-oxopropanenitrile) (**4**).

Treatment of 3,3'-(3,4-dimethylthieno[2,3-b]thiophene-2,5-diyl)bis(3-oxopropanenitrile) (**2**) [20] with sodium hydride and carbon disulfide followed by methyl iodide afforded compound **3**, which reacted with *o*-phenylenediamine in refluxing ethanol to give 3,3'-(3,4-dimethylthieno[2,3-b]thiophene-2,5-diyl)bis(2-(1H-benzo[d]imidazol-2(3H)-ylidene)-3-oxopropanenitrile) (**4**, Scheme 1). Its IR spectrum revealed two absorption bands at 3,214 and 3,177 cm^-1^ due to two NH groups and two bands at 2,195 and 1,670 cm^-1^ assignable to nitrile and carbonyl functions, respectively. The ^1^H-NMR spectrum of the reaction product displayed singlets at δ 2.49 and 13.1 due to methyls and two NH protons, respectively, in addition to an aromatic multiplet in the δ 7.28–7.59 region. The lack of an active methine proton signal in the ^1^H-NMR spectrum indicates that it exists exclusively in the 1,3-dihydrobenzimidazol-2-ylidene structure **4**, and rules out the isomeric **6**. Further evidence for the structure of compound **4** was provided by its alternative synthesis shown in Scheme 1. Thus, treatment of ester **1** [21] with an equimolar quantity of 2-cyanomethyl-1*H*-benzimidazole in the presence of sodium hydride in benzene under refluxing conditions, followed by neutralisation of the formed salt, afforded a product identical in all respects (mp., mixed mp., IR and MS spectra) with compound **4**. 

**Scheme 2:**
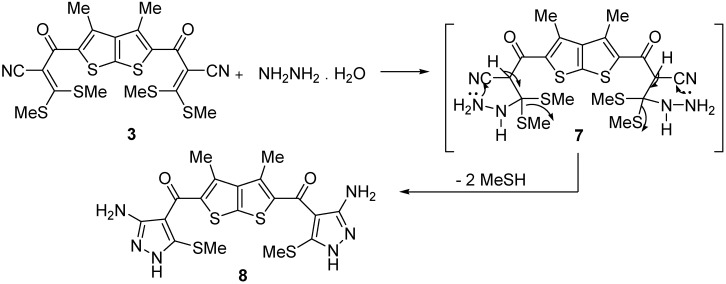
Synthesis of (3,4-dimethylthieno[2,3-b]thiophene-2,5-diyl)bis((3-amino-5-(methylthio)-1H-pyrazol-4-yl)methanone) (**8**).

Reaction of the product **3** with hydrazine hydrate gave 3,4-dimethylthieno[2,3-b]thiophene-2,5-diyl-bis-(3-amino-5-(methylthio)-1H-pyrazol-4-yl)methanone (**8**, Scheme 2). The ^1^H-NMR spectrum of compound **8** revealed two signals at δ 2.49 and 2.89 due to two CH_3_ groups, in addition to two D_2_O-exchangeable signals at δ 5.50 and 7.95 due to NH_2_ and NH protons, respectively.

**Scheme 3:**
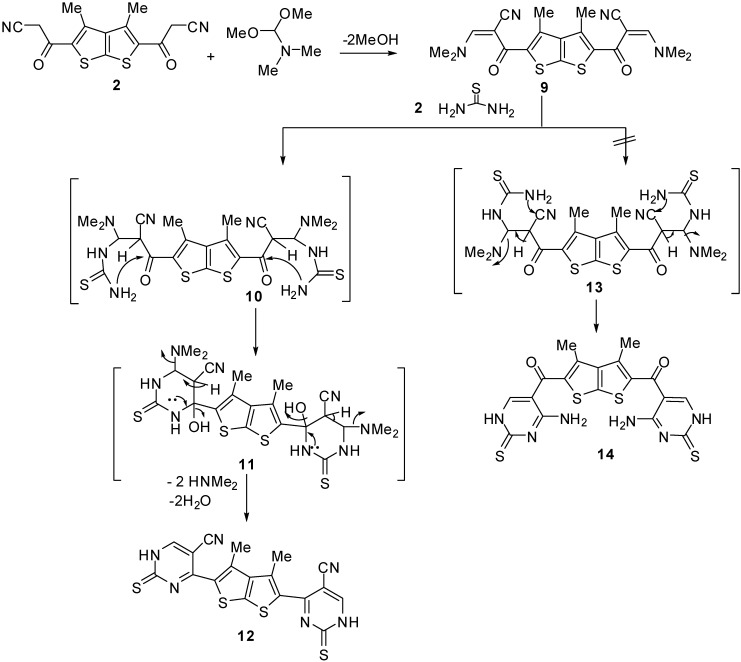
Synthesis of 4,4'-(3,4-dimethylthieno[2,3-b]thiophene-2,5-diyl)bis(2-thioxo-1,2-dihydropyrimidine-5-carbonitrile) (**12**).

Treatment of compound **2** with dimethylformamide-dimethylacetal (DMF-DMA) in refluxing xylene afforded 2-({5-[-2-cyano-3-(dimethylamino)-2-propenoyl]-3,4-dimethylthieno[2,3-b]thiophen-2-yl}carbonyl)-3-(dimethylamino)-2-propenenitrile (**9**) [20]. When compound **9** was treated with thiourea in refluxing ethanol, the novel 4,4'-(3,4-dimethylthieno[2,3-b]thiophene-2,5-diyl)bis(2-thioxo-1,2-dihydropyrimidine-5-carbonitrile) (**12**) was produced (Scheme 3). The structure of the obtained product was assigned as **12** and not the other expected derivative **14** based on its spectral data. For example, the IR spectrum of the reaction product revealed no absorption bands due to amino groups and revealed absorption bands at 3,200 and 2,210 cm^-1^ assignable to NH groups and nitrile functions, respectively. The formation of compound **12** is assumed to take place *via* nucleophilic addition of NH_2_ in thiourea to the double bond in the enaminone **9** to give the acyclic non-isolable intermediate **10**, which underwent intramolecular cyclization with subsequent aromatization *via* loss of water and dimethylamine molecules to afford the final product **12** (Scheme 3).

**Scheme 4:**
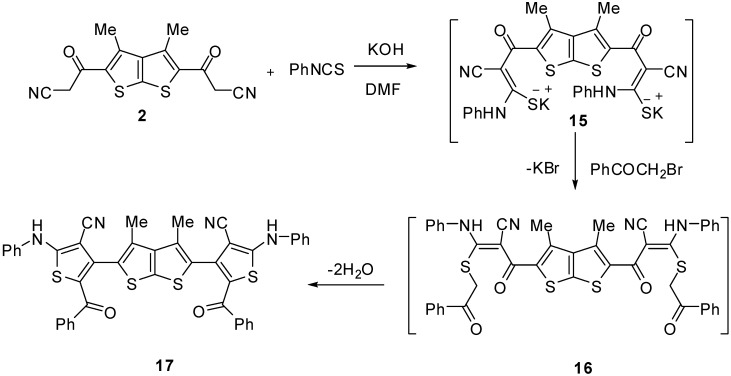
Synthesis of 4,4'-(3,4-dimethylthieno[2,3-b]thiophene-2,5-diyl)bis(5-benzoyl-2-(phenyl- amino)thiophene-3-carbonitrile) (**17**).

The nucleophilic addition of the thieno[2,3-b]thiophene **2** to phenyl isothiocyanate in DMF, in the presence of potassium hydroxide, afforded the corresponding potassium salt **15**. Heterocyclisation of the intermediate **15** with an equimolar amount of the 2-bromo-1-phenylethanone [22] furnished one isolable product (as verified by TLC analysis). The reaction product was identified as 4,4'-(3,4-dimethylthieno[2,3-b]thiophene-2,5-diyl)bis(5-benzoyl-2-(phenylamino)thiophene-3-carbonitrile) (**17**, Scheme 4). The IR spectrum of compound **17** revealed absorption bands at 1,618, 2,212 and 3,277 cm^‑1^ due to carbonyl group, nitrile and NH functions, respectively. Its ^1^H-NMR spectrum showed signals at δ 2.07 due to two CH_3_ groups and a D_2_O-exchangeable peak at δ 10.6 due to two NH protons, in addition to an aromatic multiplet in the δ 7.09–7.53 region. The aforementioned results indicate that the reaction of the intermediate **15** with 2-bromo-1-phenylethanone proceeds *via* loss of two water molecules from the non-isolable intermediate **16** (Scheme 4).

## 3. Experimental

### General

All melting points were measured on a Gallenkamp melting point apparatus. The infrared spectra were recorded in potassium bromide disks on a Pye Unicam SP 3300 or Shimadzu FT IR 8101 PC infrared spectrophotometers. The NMR spectra were recorded on a Varian Mercury VX-300 NMR spectrometer. ^1^H spectra were run at 300 MHz and ^13^C spectra were run at 75.46 MHz in deuterated dimethyl sulfoxide (DMSO-*d_6_*). Chemical shifts were related to that of the solvent. Mass spectra were recorded on a Shimadzu GCMS-QP 1000 EX mass spectrometer at 70 e.V. Elemental analyses were carried out at the Microanalytical Center of Cairo University, Giza, Egypt. 

*2-({5-[2-Cyano-3-(1,1-bis-methythio)-2-propenoyl]-3,4-dimethylthieno[2,3-b]thiophen-2-yl}carbonyl)-3-(1,1-bis-methylthio)-2-propenenitrile* (**3**). To a stirred solution of sodium hydride (0.96 g, 40 mmol) in dimethylsulfoxide (20 mL), compound **2** (3.02 g, 10 mmol) was added. The resulting mixture was stirred for 30 min, and then carbon disulfide (1.52 g, 20 mmol) was added. After 2 h of stirring, methyl iodide (5.68 g, 40 mmol) was added and the stirring was continued for additional 4 h. The resulting reaction mixture was then poured over crushed ice and the solid product was filtered off, washed with water, dried and finally recrystallised from ethanol to afford bis(methylthio)methylene derivative **3** in 60% yield, mp 170 °C; IR (KBr) ν_ max_: 1,698 (C=O), 2,210 (C≡N), 2,985 (aliphatic CH) cm^−1^. ^1^H-NMR (DMSO-d_6_): δ 2.23 (s, 6H, CH_3_), 2.53 (s, 12H, CH_3_). Anal. Calcd for C_20_H_18_N_2_O_2_S_6_(510.76): C, 47.03; H, 3.55; N, 5.48. Found: C, 47.13; H, 3.48; N, 5.40%.

*3,3'-(3,4-Dimethylthieno[2,3-b]thiophene-2,5-diyl)bis(2-(1H-benzo[d]imidazol-2(3H)-ylidene)-3-oxo-propanenitrile)* (**4**) **Method A.** *o*-Phenylenediamine (0.22 g, 2 mmol) was added to a solution of bis(methylthio)methylene derivative 3 (0.51 g, 1 mmol) in ethanol (20 mL). The mixture was refluxed for 3 h and then allowed to cool. The solid formed was filtered off, washed with ethanol and recrystallised from DMF/water to afford compound 4 in 72% yield, mp < 320 °C; IR (KBr) ν_ max_: 1,670 (C=O), 2,195 (C≡N), 3,214 and 3,177 (NH) cm^−1^; ^1^H-NMR (DMSO-d_6_): δ 2.49 (s, 6H, 2CH_3_), 7.28–7.32 (d, 4H, *J =* 8.7 Hz), 7.56–7.59 (t, 4H, *J =* 8.7 Hz), 13.1 (s, 4H, D_2_O-exchangeable, 4NH); ^13^C-NMR (DMSO-d_6_): δ 8.3 (2CH_3_), 111.0 (2=C-C), 115.3 (2CN), 117.6, 118.9, 142.2, 156.0 (benzimidazole ArC), 130.4, 131.8, 138.8, 140.1, (thienothiophene ArC), 185.9 (2C=O). Anal. Calcd for C_28_H_18_N_6_O_2_S_2_ (534.61): C, 62.91; H, 3.39; N, 15.72. Found: C, 62.81; H, 3.32; N, 15.67%.

**Method B.** To a mixture of diethyl 3,4-dimethylthieno[2,3-b]thiophene-2,5-dicarboxylate (**1**, 3.12 g, 10 mmol) and 2-(1H-benzo[d]imidazol-2-yl)acetonitrile (3.14 g, 20 mmol) in dry benzene (25 mL) and dimethylformamide (1 mL) was added sodium hydride (0.96 g, 60%). The reaction mixture was refluxed for 4 h, then allowed to cool. The solid that precipitated was collected by filtration, washed with ether and dried. The solid product was dissolved in water and the resulting solution was neutralised to pH 7 with concentrated hydrochloric acid. The precipitated solid was collected by filtration, washed with water and dried. Recrystallisation of the crude product from DMF/water gave a product (60% yield) identical in all respects (TLC, IR spectrum) with that obtained by method A.

*Synthesis of (3,4-dimethylthieno[2,3-b]thiophene-2,5-diyl)bis((3-amino-5-(methylthio)-1H-pyrazol-4-yl)methanone)* (**8**). To a solution of compound **3** (0.51 g, 1 mmol) in EtOH (20 mL), hydrazine hydrate (80%, 0.2 mL, 2 mmol) was added and the reaction mixture was refluxed for 4 h, and then left to cool. The solid product so formed was filtered off, washed with EtOH and dried. Recrystallization from DMF/ EtOH afforded **8** in 55% yield; mp 302 °C; IR (KBr) ν_ max_: 3,427, 3,214 and 3,177 (NH, NH_2_), 1,675 (C=O) cm^−1^; ^1^H-NMR (DMSO-d_6_): δ 2.49 (s, 6H, 2CH_3_), 2.89 (s, 6H, 2CH_3_), 5.5 (s, 4H, D_2_O-exchangeable, 2NH_2_), 7.95 (s, 2H, D_2_O-exchangeable, 2NH); ^13^C-NMR (DMSO-d_6_): δ 8.3 (2CH_3_), 11.7 (2CH_3_-SH), 87.8, 115.3, 142.2, (pyrazole ArC), 130.4, 131.8, 136.3, 140.1, (thienothiophene ArC), 185.9 (2C=O). Anal. Calcd for C_18_H_18_N_6_O_2_S_4_(478.63): C, 45.17; H, 3.79; N, 17.56. Found: C, 45.27; H, 3.86; N, 17.51%

*Synthesis of 4,4'-(3,4-dimethylthieno[2,3-b]thiophene-2,5-diyl)bis(2-thioxo-1,2-dihydropyrimidine-5-carbonitrile)* (**12**). To a mixture of compound **9** (0.41 g, 1 mmol) and thiourea (0.15 g, 2.0 mmol) in ethanol (30 mL), a few drops of piperidine was added and the reaction mixture was refluxed for 8 h, then left to cool to room temperature. The precipitated product was filtered off, washed with EtOH, dried and finally recrystallized from DMF to afford compound **12** in 78% yield; mp. 318 °C; IR (KBr) 3,200 (NH), 2,210 (C≡N), cm^−1^; ^1^H-NMR (DMSO-d_6_): δ 2.49 (s, 6H, 2CH_3_), 6.37 (s, 2H, 2CH pyrimidine), 11.62 (s, 2H, D_2_O-exchangeable, 2NH). Anal. Calcd. for C_18_H_10_N_6_S_4_(438.57): C, 49.29; H, 2.30; N, 19.16. Found: C, 49.20; H, 2.22; N, 19.106%.

*Synthesis of 4,4'-(3,4-dimethylthieno[2,3-b]thiophene-2,5-diyl)bis(5-benzoyl-2-(phenylamino)- thiophene- 3-carbonitrile)* (**17**). To a stirred solution of potassium hydroxide (0.11 g, 2 mmol) in DMF (20 mL) was added compound **2** (0.30 g, 1 mmol). After stirring for 30 min, phenyl isothiocyanate (0.27 g, 2 mmol) was added to the resulting mixture. Stirring was continued for 6 h, and then 2-bromo-1-phenylethanone (0.40 g, 2 mmol) was added portionwise over a period of 30 min. After the addition was complete, the reaction mixture was stirred for additional 12 h, during which the 2-bromo-1-phenylethanone went into solution and a yellow product precipitated. The solid product was filtered off, washed with EtOH and dried, Recrystallization from EtOH/DMF afforded **17** in 86% yield, mp < 320 °C; IR (KBr) ν_ max_: 3,277 (NH), 2,212 (C≡N), 1618 (C=O) cm^−1^; ^1^H-NMR (DMSO**-**d_6_): δ 2.07 (s, 6H, 2CH_3_), 7.09–7.53 (m, 20H, ArH), 10.6 (s, 2H, D_2_O-exchangeable, 2NH); ^13^C-NMR(DMSO-d_6_): δ 8.3 (2CH_3_), 115.3 (2CN), 116.3, 117.6, 118.9, 125.3, 126.6, 127.9, 129.1, 136.7 (ArC’s), 130.4, 131.8, 138.8, 140.1 (thienothiophene ArC), 111.0, 133.3, 145.8, 156.0 (thiophene ArC), 187.9 (2C=O). MS *m/z* (%) 774 (M^+^+1, 88.6%), 773 (M^+^, 100%), 351 (80.6%), 212 (16.2%), 63(30.3%). Anal. Calcd for C_44_H_28_N_4_O_2_S_4_ (772.98): C, 68.37; H, 3.65; N, 7.25. Found: C, 68.31; H, 3.69; N, 7.20%.

## 4. Conclusions

In summary, the reactivity of diethyl 3,4-dimethylthieno[2,3-b]thiophene-2,5-dicarboxylate (1) as a versatile and readily accessible building block for the synthesis of new bis-heterocycles incorporating thieno[2,3-b]thiophene moieties of potential biological and pharmaceutical importance was investigated.
